# Health System Determinants of Delivery and Uptake of HPV Vaccination Services Among Involuntary Migrant Populations: A Qualitative Systematic Review

**DOI:** 10.3390/vaccines13101064

**Published:** 2025-10-18

**Authors:** Jennifer Nyawira Githaiga, Jill Olivier, Susanne Noll, Edina Amponsah-Dacosta

**Affiliations:** 1 Division of Social and Behavioural Sciences, School of Public Health, Faculty of Health Sciences, University of Cape Town, Cape Town 7925, South Africa; 2 Health Policy and Systems Division, School of Public Health, Faculty of Health Sciences, University of Cape Town, Cape Town 7925, South Africa; 3 Vaccines for Africa Initiative, School of Public Health, Faculty of Health Sciences, University of Cape Town, Cape Town 7925, South Africa

**Keywords:** adolescents, cervical cancer, human papillomavirus vaccination, migrants, health system, policy

## Abstract

**Background:** Migrant populations are commonly under-immunised relative to general populations in host countries. The evidence base on routine vaccination among migrant children suggests that higher priority is given to infants and younger children compared to adolescents. Though migrants are often classified as a homogenous group, different sub-populations of migrants exist, including voluntary migrants who choose to move and involuntary migrants forcibly displaced by humanitarian crises. The human papillomavirus (HPV) vaccine, a relatively recent addition to global routine immunisation schedules for adolescents, is a useful proxy for understanding vaccine equity for this under-prioritised group. This qualitative systematic review explores health system determinants of delivery and uptake of HPV vaccination services among involuntary migrants. **Methods:** A literature search was conducted across ten electronic databases. An analytical framework tailored to the migrant context aided in capturing the complexity and magnitude of systemic factors that determine vaccine delivery and uptake among involuntary migrants. Of the 676 records retrieved, 27 studies were included in this review. **Results:** Key determinants of vaccine delivery include adaptation of immunisation policies for migrant inclusiveness, implementation of migrant-targeted interventions, health provider recommendations, electronic health records, and free vaccines. Uptake determinants include access dependent on legal status, awareness-related determinants akin to culturally appropriate health messaging, and acceptance-related determinants associated with sociocultural beliefs, misinformation, and distrust. **Conclusions:** Prioritising vaccination programmes linked with non-outbreak-related diseases is challenging in the disruptive context of humanitarian crises given fragile health systems, limited resources, loss of health infrastructure and deployment of health personnel to emergency care. We strongly advocate for global actors at all health systems levels to actively reform national HPV vaccination programmes to enhance inclusivity of adolescent girls in crises settings or resettled in host countries.

## 1. Introduction

Globally, cervical cancer is the fourth most frequently occurring women’s cancer, with approximately 660,000 new cases and 350,000 deaths reported worldwide in 2022 [1]. In several countries, predominantly low- and middle-income countries (LMICs) in sub-Saharan Africa, Southeast Asia, and South America, cervical cancer is the most frequently occurring disease and the top cause of cancer-related deaths among women [1]. Women in LMICs are disproportionately impacted by cervical cancer [2,3]. In 2018, 84% of cervical cancer incidence and 87–90% of cervical cancer mortality occurred in LMICs [3]. Further, the burden of cervical cancer is greater among younger women globally and specifically in LMICs; for instance, in 2020, while 20% of total cancer-related deaths among women occurred due to cervical cancer, of these deaths, about 15% were aged 30–49 years [4]. In Southern and Eastern Africa, most cancer-related deaths among women were due to cervical cancer [4]. Higher cervical cancer incidence rates and lower adherence to cervical cancer prevention measures have been reported among migrant women residing in high-income countries (HICs), mainly in Europe, compared to local women [5]. A study on cancer among migrants in Sicily, Italy, documented higher cervical cancer incidence rates coupled with a higher odds ratio among migrants compared to the general population [6]. A Norway-based study observed that 52% of migrant women had not undergone cervical cancer screening and that the likelihood of not adhering to screening was 1.72 times higher among migrant women compared to native Norwegian women [7].

The leading cause of cervical cancer is incessant infection with high-risk human papillomavirus (HPV), which is sexually transmitted and accounts for over 90% of all cervical cancer cases worldwide [3,8], particularly among women aged under 35 years [3]. As an infection-related cancer, cervical cancer can be prevented through HPV vaccination, a proven cost-effective intervention in several countries worldwide [8,9]. The World Health Organization (WHO) recommended routine vaccinations for adolescents including hepatitis B, tetanus-diphtheria-acellular pertussis booster, rubella, and HPV series [10]. The HPV vaccine is a relatively recent addition and may serve as a proxy for understanding immunisation programmes for adolescent migrants. Though HPV is not associated with vaccine-preventable disease (VPD) outbreaks, curative cervical cancer management, particularly in low- and middle-income contexts where women develop cervical cancer during their most industrious years, may not only burden health systems but may also negatively impact national economies [11]. The WHO 90-70-90 strategy to eradicate cervical cancer as a global public health threat by 2030 [12] adopts a life course approach with three main targets: primary prevention through HPV vaccination of 90% of girls by the age of 15 years, secondary prevention through screening 70% of women at 35 years and at 45 years, and tertiary prevention through treatment of pre-cancerous lesions and management of invasive cervical cancer among 90% of affected women [12]. Evidence demonstrates inequitable availability and adoption of HPV vaccination geographically in the WHO regions [2,9]. By mid-2020, 85% of countries in the Americas and 77% of European countries offered HPV vaccine through their national immunisation programmes (NIPs), in contrast to LMICs where the vaccine was available in 31% of African countries and 40% of Asian countries [13].

Migration is propelled by various factors, especially economic and political ones [14,15]. Though migrants are often classified as a homogenous group, different sub-populations of migrants exist, including persons who voluntarily choose to relocate to a new country of residence (voluntary migrants), which are different from persons forcibly displaced by war and other humanitarian crises (involuntary migrants) [16,17] (Box 1). Migrants may have some common needs and experiences, but there are also variances evident among different migrant sub-populations. For example, involuntary migrants may have limited access to immunisation services during conflict [18,19] and while awaiting determination of legal status in host countries [20,21] compared with voluntary labour migrants who may have more access [22,23,24]. Regarding routine immunisation (including HPV vaccination), all types of migrant populations are mostly under-immunised relative to the general population in host countries [20,21,25]. This trend was highlighted during the COVID-19 pandemic, with reports of migrants being excluded from accessing COVID-19 vaccination based on their migration status—for example, Venezuelan migrants who fled to Latin America due to socio-political and economic instability [26]. Under-immunisation of migrants has been linked to upsurges of VPDs among migrant and general populations in various regions including some European countries and the United States of America (USA) [27,28]. While there is a lot of focus on migrants in HICs, in fact, a significant proportion of migrants are hosted in LMICs. For example, it was estimated that, in 2022, 76% of the world’s refugees and other people requiring international protection were being hosted in LMICs [29].

Box 1 **Typology of migrant sub-populations.**

**Voluntary migrant:** A person who chooses to relocate from their usual place of residence to a different location either within or outside of their country’s borders, e.g., labour migrants and international students.

**Involuntary migrant/forcibly displaced:** A person forcibly displaced within or outside of their country’s border due to war, persecution, and/or other humanitarian crises. This includes the following:

⮚ *Asylum seeker*—a person seeking protection in a country outside of their home country but whose status is yet to be determined by the host country.
⮚ *Refugee*—a person who is forced to leave their home country to seek international protection due to armed conflict, persecution, and/or other humanitarian crises.
⮚ *Internally displaced person*—a person forced out of their home who moves to a different location within their home country.
⮚ *Unaccompanied and separated child/minor*—a person aged 18 years and below who has been separated from both parents and other legal guardians.

It has been widely suggested that the main barriers to migrants accessing vaccination in LMICs are systemic in nature [17,20,21]. These might be loosely divided into health system hardware and software factors. Structural factors related to health system hardware, which may impact vaccination service provision among migrant populations, include staff shortages, lack of clear policies, out-of-pocket costs for vaccination in the absence of free vaccination services, and geographic/physical access to services [17,20]. Less-tangible health system-related factors, referred to as software [30], include migrants’ (mis)trust of vaccines, host governments and attendant healthcare systems, socio-cultural and religious beliefs, fear of discrimination based on legal status, and knowledge gaps linked to language and literacy barriers [20,21]. In this manuscript, the term ‘determinants’ is used as an umbrella term with reference to the array of factors—including physical, social, economic, environmental, and systemic factors—that influence health experiences and outcomes of individuals and populations [31]. Health system hardware determinants play a key role in vaccination service delivery, while software determinants have an influential role in determining uptake and more so, given the vulnerability of migrants linked to their status in host countries.

Applying a health systems lens entails comprehending the dynamism and complexity of interconnected, interdependent relationships between hardware and software and viewing the various parts as components of the health system as a whole [32]. Understanding these health system determinants is a prerequisite to addressing vaccination-related disparities faced by migrant populations and ultimately, achieving the Immunisation Agenda 2030 goal of full vaccine access to all [33].

This qualitative systematic review was conducted to explore the question: what are the health system determinants of delivery and uptake of HPV vaccination services among involuntary migrant populations? The main objectives of this review were to describe health system determinants of delivery and uptake of HPV vaccination services among involuntary migrant populations and to enhance our comprehension of how these determinants may facilitate or impede provision and utilisation of migrant-inclusive HPV vaccination services among involuntary migrant populations. Based on our findings, we proposed recommendations for research, policy, and practice for key health system actors to support existing and future migrant-inclusive HPV vaccination services for involuntary migrant populations.

## 2. Materials and Methods

This systematic review follows the Joanna Briggs Institute (JBI) guidelines for evidence synthesis [34] and the preferred reporting items for systematic reviews and meta-analyses (PRISMA) 2020 guidelines, found in Data S1 [35]. Whereas it is increasingly becoming best practice to register systematic reviews, the JBI guidelines recommend but do not require prospective registration of protocols, and, as such, we did not register our protocol. A published scoping review was conducted as a preamble to this systematic review [36], which details the processes and decisions leading to this systematic review, serves as a priori protocol in keeping with the JBI stipulations for qualitative evidence syntheses [34].

## 3. Search Strategy

The search strategy was developed in close consultation with an information specialist who guided the process of identifying relevant literature sources. The search, informed by a scoping review [36], was optimised by identifying key search words and variations in these including synonyms, using truncations, Boolean operators, and medical subject headings (MeSH) terms, as appropriate, and optimising these across multiple databases. Search terms included the following: population descriptors (Migrants OR “asylum seekers” OR displaced OR refugee OR transients OR undocumented OR “forcibly displaced” OR resettled OR “persons-of-interest” OR “internally displaced persons” OR irregular OR illegal OR “illegal immigrant” OR aliens OR “foreign-born” OR “internationally displaced person” OR “unaccompanied child*” OR “unaccompanied minor” OR “separated child*” OR “involuntary migrant” OR “stateless person”); intervention descriptors (“Human papillomavirus vaccin*” OR “HPV vaccin*” OR “human papillomavirus immuni*”), and outcome descriptors (“health care provider” OR “health provider” OR “health policy” OR Coverage OR uptake OR quality OR availability OR acceptability OR awareness OR access OR “service provision” OR “service delivery” OR affordability OR “healthcare cost” OR “cost effectiveness” OR “healthcare provision” OR efficiency OR safety OR “financial risk protection” OR equity OR responsiveness OR “health outcomes” OR “health system” OR efficacy).

We developed a comprehensive search strategy for PubMed which was then adapted for other databases. We searched PubMed, Web of Science, Scopus, and EBSCOHost (Academic Search Premier, Africa-Wide Information, Cumulative Index to Nursing and Allied Health Literature [CINAHL], Health Source Nursing/Academic Edition, Health Source Consumer Edition, APA PsycArticles and APA PsycInfo) databases. The search strings per database are documented in Data S2. Manual searches of reference lists of articles and a supplementary search on Google Scholar were conducted to look for additional sources. No date limitations were applied to enhance the scope of our literature search. The preliminary search was conducted on 24 October 2023. Furthermore, an updated search was conducted on 28 June 2025.

## 4. Eligibility Criteria

Primary empirical qualitative, quantitative, and mixed-methods studies featuring HPV vaccination experiences among involuntary migrants (either as the main focus or as a sub-population of migrants) were included as evidence sources. The inclusion and exclusion criteria, based on the Population-Intervention-Comparison-Outcomes-Study design (PICOS) tool frequently employed in qualitative systematic reviews [37], are summarised in Table 1.

## 5. Screening and Study Selection

Records were exported from the various databases to EndNote [38] to screen for duplicates, which were removed. The records were then exported to the Rayyan online management platform [39] for further screening to eliminate any additional duplicates detected. This was followed by title and abstract screening completed in Rayyan. Next, full texts were retrieved and reviewed, and those meeting the eligibility criteria were selected for inclusion. Two reviewers independently screened study articles, and discrepancies were resolved by consensus. When disagreement persisted, a third reviewer was consulted.

## 6. Data Extraction

The design and development of the data extraction tool were guided by our preliminary scoping review [36]. Core variables included contextual information related to the circumstances of forced displacement, health system facilitators and barriers to HPV vaccine uptake and delivery, and outcome indicators of robust health systems. Two co-authors, JNG and EAD, conducted data extraction while JO provided oversight and arbitration in instances where conflicts arose during decision making. All three authors participated in the iterative process leading to the items included in the data extraction sheet, auditing the pilot data extraction sheet, and making recommendations for further enhancement, discussions about emerging data following a pilot data extraction exercise using a few studies, reviewing the final revised data extraction sheet, and discussions around emerging themes following completion of data extraction.

## 7. Data Analysis and Synthesis

We developed an analytical framework drawing from several existing frameworks (Table 2). Given that immunisation service provision occurs within the broader context of the health system, exploring determinants of HPV vaccination delivery and uptake among migrant populations necessitates understanding these determinants in conjunction with health system factors and how these interact in shaping attendant vaccination outcomes. Of the various frameworks utilised by scholars to explain vaccine-related behaviour, we drew from two frameworks deemed suitable for addressing our review objectives, namely, the P3 Model and the 5As Framework [40,41]. The P3 Model explores three key influences—patient, provider, and practice—and how these interact and shape preventive care service provision, including immunisation services [40]. This framework has been used previously by a study conducted in the USA which investigated the factors shaping HPV vaccine decision making among Vietnamese migrant mothers [42]. The 5As Framework posits that vaccine uptake is influenced by a complex array of structural, demographic, and socio-behavioural factors, namely, the 5As: access, affordability, awareness, acceptance, and activation [41]. Four of the 5As resonate with facilitators and barriers of vaccination among refugees and migrants documented in the 2022 WHO Global Evidence Review on Health and Migration (GEHM) series report [17].

Two health system frameworks were included in the analytical frame applied in this review. First, the WHO framework, comprising six fundamental health system building blocks, namely, leadership and governance, health service delivery, health workforce, health financing, health information systems, and medical products, vaccines, and technologies [43]. This framework served as a lens to explore health system factors that facilitate or impede vaccination delivery among migrant populations. Second, the Witter et al. Health Systems Strengthening Framework proposes a ‘theory of change approach’ focusing on effects that cut across several building blocks, attendant processes, and potential outcome indicators of strong health systems [44]. Drawing from the four frameworks, we developed an analytical framework specifically adapted for our review (Table 2). Information derived from the analytical model was captured in our data extraction sheet and further synthesised, culminating in the narrative data presented in the Results Section. We employed the JBI meta-aggregation approach to qualitative synthesis which entails synthesising data from multiple studies, to yield statements to inform policy and practice, in contrast to meta-ethnography which involves re-engaging with and re-interpretation of findings from individual primary studies [34].

## 8. Quality Assessment

Quality assessment was conducted using the JBI critical appraisal tools [45,46,47], and the Mixed Methods Appraisal tool [48]. The three JBI appraisal tools used were the qualitative tool, a 10-item checklist to appraise qualitative studies [45], cohort tool, an 11-item checklist to appraise cohort studies [46], and cross-sectional tool, an 8-item checklist to appraise cross-sectional studies [47]. The Mixed Methods Appraisal Tool is a 7-item checklist used to appraise mixed-methods studies. Summaries of appraisals of each of the four study genres included in this study are presented in the Results Section.

## 9. Results

**Figure 1:**
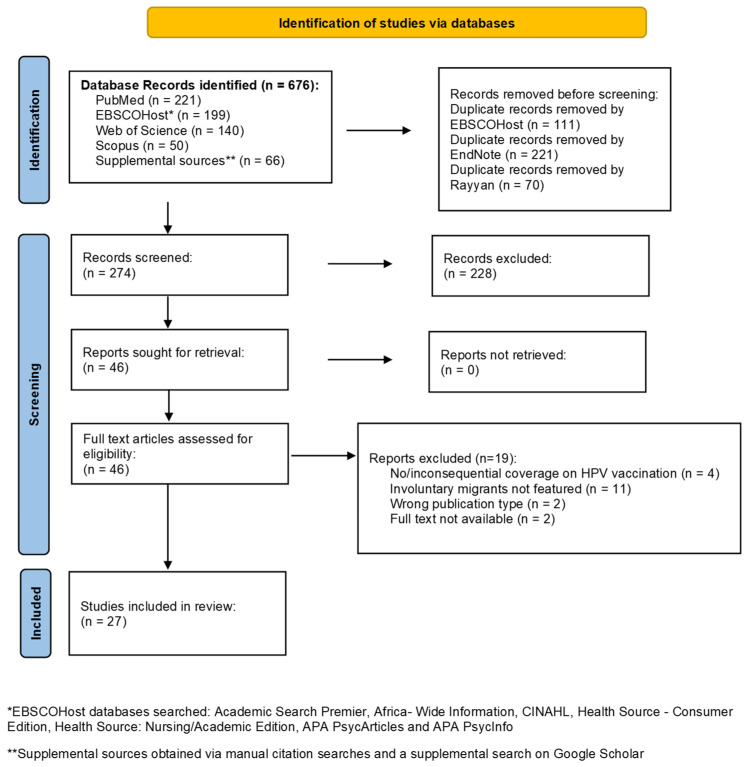
PRISMA flow chart summarising identification and screening of included studies.

The systematic search yielded a total of 676 articles, of which 274 were screened. Of these, 228 articles were excluded because they did not meet the inclusion criteria, while 27 articles meeting all inclusion criteria were selected (Figure 1).

## 10. Descriptive Characteristics

Descriptive characteristics of the studies included are summarised in Table 3. Included items were descriptively analysed according to publication dates, study design, classification of migrants, home countries, host countries, and health system actors featured. The studies were published between 2009 and 2025 with 13/27 studies (48%) published between 2020 and 2025. Of the 27 studies, 10/27 utilised qualitative designs [49,50,51,52,53,54,55,56,57,58], 9/27 were cross-sectional studies [59,60,61,62,63,64,65,66,67], 6/25 were cohort studies [68,69,70,71,72,73], while 2/27 utilised a mixed-methods study design [74,75]. One mixed-methods item [74] and a cross-sectional item [62] reported on the same study, while the other mixed-methods study [75] was a sequel to a qualitative item [56]. The most widely studied category of involuntary migrants was refugees, who were the sole focus of 15/27 studies, while 2/27 Australia-based studies focused on both refugees and asylum seekers [58,73]. Of the 8/27 studies that used the classification ‘refugees and migrants’, 4/27 studies used this as a blanket term with no distinction between refugees and migrants [51,55,61,65], while 4/27 studies (all Canada-based) included refugees as a sub-population of migrants [53,54,56,75], while 2/27 studies distinguished between voluntary and involuntary migrants [66,68].

**Figure 2:**
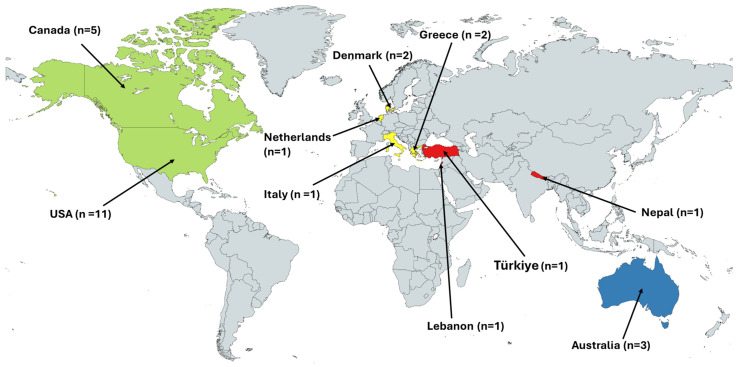
Countries hosting migrants from the 27 included studies. (created with mapchart.net).

The majority of involuntary migrants featured were from LMICs and resettled mostly in HICs, except for three studies conducted among migrants residing in LMICs, namely, Nepal [59], Lebanon [63], and Türkiye [67]; the rest of the studies (24/27) were from HICs (Figure 2). Over half of the included items (15/27) featured migrants resettled in North America, namely, the United States of America (USA) [49,50,51,52,61,62,64,69,70,71,74] and Canada [53,54,56,75]. There were six studies on migrants resettled in European countries, namely, Denmark [68,72], Greece [60,66], the Netherlands [57], and Italy [65]. Two studies conducted among migrants resettled in Australia [58,73], while another study presented data on migrants resettled in Australia and Canada [55]. Health system actors featured in the reviewed studies include parents and guardians (9/27), adolescents (2/27), health providers (1/27) and a broader range of migrants classified as women, who were involved in HPV vaccination decision-making processes (13/27). Mixed samples of adolescents/young adults and caregivers were featured in 2/27 studies.

## 11. Appraisal of Included Studies

**Figure 3:**
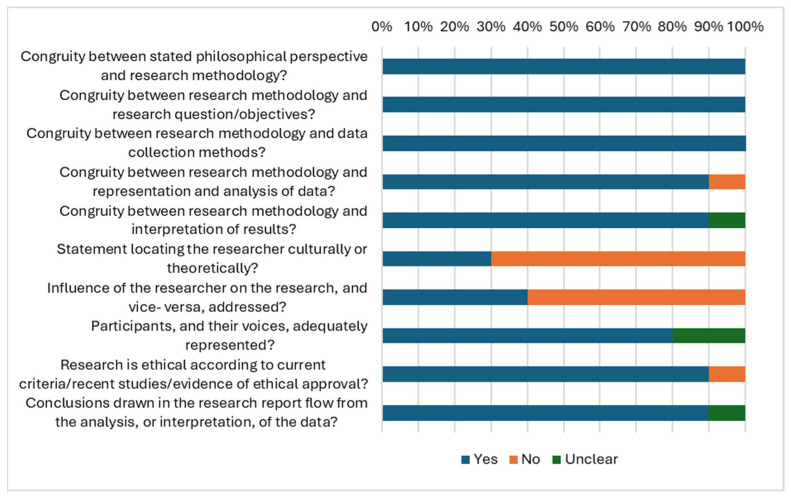
JBI summary appraisal of qualitative studies.

Primary qualitative studies (10/27) were assessed using the JBI appraisal checklist for qualitative studies [45] (Figure 3). Reflexivity was notably absent in many studies, in which there was neither mention of the researchers’ cultural or theoretical leaning (7/10) nor evidence of consideration of how researchers’ presence might have influenced data collection (6/10). Two less common limitations were lack of clarity between methods and analysis and omitting a statement on ethical procedures followed in the research. However, the study that did not explicitly include an ethics statement reported longstanding collaboration in health-related research between the university research group and the community under study, including community leaders participating as interviewers [51], implicitly implying consent to conduct the study.

**Figure 4:**
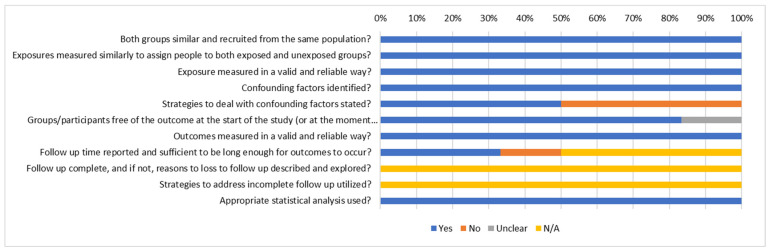
JBI summary of cohort studies.

The JBI checklist for cohort studies [46] was used to appraise the quality of the 6/27 included cohort studies (Figure 4). The main limitation identified was insufficiencies in explanations of how confounding factors were addressed in half of the studies. One study did not report adequately on follow-up time, making it difficult to establish if this time duration was ample for generation of anticipated outcomes.

**Figure 5:**
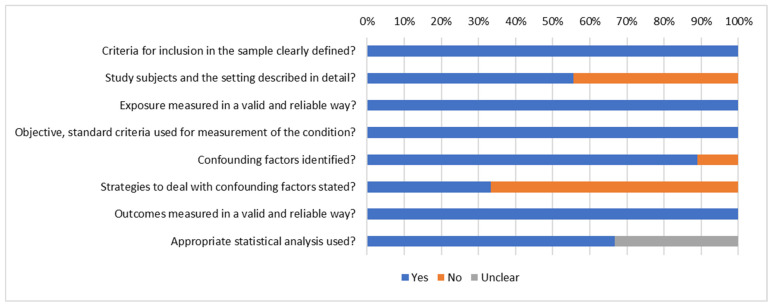
JBI summary of cross-sectional studies.

The JBI checklist for cross-sectional studies [47] was used to appraise the quality of the included 9/27 cross-sectional studies (Figure 5). Of these, 4/9 studies did not provide adequate descriptions of the research subjects and/or the research context. Further, though 8/9 studies identified confounding factors, 6/9 of the studies did not explain how they dealt with such confounders.

**Figure 6:**
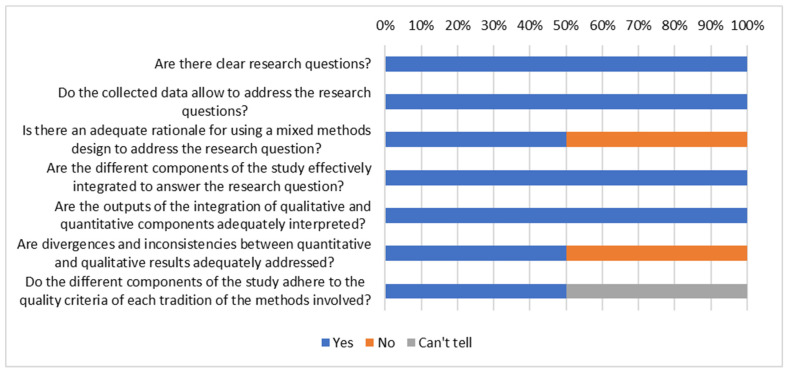
Summary appraisal of mixed-methods studies.

The Mixed Methods Appraisal Tool [48] was used to appraise the quality of the 2/27 mixed-methods studies included (Figure 6). The first limitation was lack of clarity on the rationale for conducting a mixed-methods study. The second limitation was not addressing methodological discrepancies between qualitative and quantitative results.

All 27 studies were included, notwithstanding their methodological quality, because insights gleaned from these studies informed the results.

## 12. Health System Determinants of HPV Vaccination Service Delivery

The WHO building blocks [43] were used to map health system hardware-related determinants of HPV vaccination service delivery. Determinants were grouped into those that enable and those that impede service delivery (Table 4).

Leadership and governance-related factors include policies prioritising migrants’ health needs, governance systems and the role of governments in vaccination policy immunisation. In Australia, the Queensland government endorsed the Refugee Health and Wellbeing Policy and Action Plan 2017–2020 to specifically address refugees’ and asylum seekers’ needs [73]. Also noteworthy is the Health Care Consent Act which allows children in Ontario Province, Canada, to receive HPV vaccination without parental consent [56]. Three notable policy HPV-related adaptations were identified in the USA, the first country to introduce primary care-based HPV vaccination in 2006. These adaptations include the following: (i) an initial attempt at mandatory vaccination for all grade six girls which failed in 2007; (ii) a 2008 directive making HPV vaccination mandatory for all incoming immigrant girls aged 11–26 years, which was vetoed by the National Coalition for Immigrant Women’s Rights, causing the government to recant at the end of 2009; and (iii) the current recommendation of free routine HPV vaccination for all (including migrants) aged 11–12 year olds, and as well as catch up vaccination up to 26 years of age [50,71]. In countries with decentralised governance systems such as Australia, the USA and Canada, variations in policy implementation at federal level may facilitate or impede service delivery at practice level. For instance, some provinces in Canada offer free HPV catch-up vaccination to all, including migrants, in contrast to provinces where HPV vaccination at own cost applies to individuals deemed ineligible for free publicly funded HPV vaccination services [56].

This links with the WHO financing building block. Free HPV vaccination for all via NIPs and other supporting programmes, in countries such as the Netherlands [57], the USA [50,51,71] and parts of Canada [53], enables service delivery among involuntary migrants. The cost of HPV vaccines may impede service delivery in contexts where there was either partial or no coverage. For instance, in Denmark free immunisation services (including HPV vaccination) for all eligible legally registered citizens and permanent citizens include refugees but exclude asylum seekers and other newly arrived migrants whose status is yet to be determined [68], while in Lebanon HPV vaccination is available at own cost [63]. Government-endorsed supporting programmes such as the Vaccines for Children Program and the Advisory Committee on Immunisation Practices in the USA [50,69,71] and the Centre for Infectious Disease Control in the Netherlands [57] serve as implementers of immunisation-related policies and programmes.

Service delivery is contingent on availability of HPV vaccination programmes and the various modes of implementation of these programmes, which determine whether HPV vaccines reach involuntary migrants. In the WHO European region, 37 out of 53 countries include HPV vaccination in their NIPs [66]. For instance, Greece provides free HPV vaccination through its Ministry of Health for all 12–16-year-old girls [66]. Lack of HPV vaccination programmes in migrants’ home countries impedes access to such services as observed among refugees, predominantly from LMICs, resettled in the USA [70] and Syrian refugees resettled in Greece [60]. Modes of HPV vaccination implementation include school-based programmes, catch-up vaccination and other migrant-targeted interventions, integrated services and public–private partnerships. In Denmark, HPV vaccination was available at a cost until 2009, when the vaccine was added to the national routine immunisation programme for girls and boys aged 12–18 years old, with a supplementary catch-up programme at no cost for all registered persons including migrants [68,72]. In Canada, Saskatchewan Province has a publicly funded, school-based HPV vaccination programme for all grade 6 children and catch-up specialised HPV vaccination public health immunisation clinics for all 9–26-year-olds [53]. School-based programmes may impede service delivery by limiting HPV vaccine-eligible age groups, for example, in the Netherlands where services are limited to 12-year-olds only [57]. Vaccination service delivery may also be impeded by place of residence, for example, Syrian refugees in Lebanon, most of whom do not reside in refugee camps and thus may miss out on vaccination opportunities [63]. Health-related messaging may also impede vaccination service delivery by targeting a narrow audience such as younger women, instead of women of all age groups, as observed in a study of Somali and Mexican immigrants and refugees based in Ohio, USA [61].

The Refugee Health Assessment Program (RHAP) of Massachusetts Department of Public Health is an example of a USA-based intervention specifically targeting involuntary migrants [69] that enables service delivery to this population. Even so, RHAPs are not standardised in the USA, so there may be variances in implementation and consequently, RHAP access for migrant populations may vary in different states [69]. Due to ensuing financial crisis in Greece, primary healthcare is facilitated via public–private partnerships between non-governmental organisations (NGOs) and the government, through NGO-run polyclinics which provide free HPV vaccination to all vulnerable populations regardless of legal or other status, including refugees and migrants [66]. In Australia, HPV vaccination in Queensland is offered through the refugee clinic model, a collaborative venture involving the Partnership Advisory Group Queensland, local primary health networks, and the Refugee Health Community Advisory Group, comprising health development consultants from different refugee backgrounds [73]. Another intervention strategy featured in a study of refugees and migrants in Vancouver, Canada, and Sydney, Australia, is integrating HPV vaccination into the broader range of sexual and reproductive services [55]. However, this study noted that integrated service delivery did not necessarily correlate with improved uptake due to other socio-cultural factors which are discussed in the section on determinants of vaccination uptake.

Routine vaccinations may be categorised as mandatory, such as those required for school admission in countries like Canada [56] and Lebanon [63], or voluntary. This may have implications for service delivery in vaccines, including HPV, which are categorised as routine voluntary vaccines. For instance, in Lebanon, non-mandatory vaccines including HPV are excluded from the Lebanese National Immunisation calendar and only available at one’s own cost [63], as different from Greece where all vaccinations are voluntary but available free via the NIP [66]. In certain contexts, the distinction between routine mandatory and voluntary vaccinations may be less apparent, for instance, in the USA where HPV vaccination, though voluntary, is included in the RHAP routine immunisation package for all incoming refugees [69]. HPV vaccine service delivery via the RHAP was associated with increased vaccination coverage in Massachusetts where higher uptake of the first HPV vaccine dose was reported among 13–17-year-old refugees (68%) compared to adolescents of the same age in the general population (45%) [69].

Within the health workforce, it is worth noting the role of health providers as gatekeepers in enabling HPV vaccination by recommending the vaccine and as the main sources of HPV vaccination-related information, as reiterated in several studies, including some conducted among refugees resettled in the USA [49,50,52] and a group of refugees and migrants in Italy [65]. Health providers also play key roles in administering the HPV vaccine, including obtaining informed consent from parents and guardians of minors as documented in a Canadian study [53]. A sequel of two included articles that form part of the same Canadian-based study suggested two possible reasons for lack of vaccine recommendations by health providers, which may impede service delivery. First, health providers were often too busy to discuss and recommend HPV vaccination to newly resettled migrants, a view shared by both patients (recent migrants) and health professionals [56,75]. Health professionals clarified that, in their immunisation-related conversations, precedence was given to routine mandatory vaccinations required for school entry at the expense of non-mandatory vaccines such as HPV [56]. The second reason given for health professionals’ reticence to recommend HPV vaccination to new migrants was the perception that the cost of CAD 540 was prohibitive for those not eligible for publicly funded HPV vaccination and those without health insurance [56]. Further to this, a study conducted in Denmark also noted that health professionals were typically ill-prepared to serve migrants, underscoring the need for health professionals to be cognisant of the heightened risk of non-immunisation among certain migrant sub-populations such as refugees [72].

Health information systems in the form of electronic records featured in five of the articles reviewed. These studies underscored the need to mobilise resources by synchronising immunisation data for quality service delivery as well as production of data that may be used in research aimed at informing immunisation policy and practice. Establishing the immunisation status of Syrian refugees residing in Lebanon proved problematic in the absence of electronic health records for children who had no documentation of prior and/or current immunisation and where parental recall of children’s immunisation status was hampered by low literacy levels [63]. Health providers of recently arrived immigrants in Canada recommended a centralised electronic vaccination database for easy access to migrants’ records, to avoid both over- and under-immunisation [56]. Further, the health providers advocated for incorporation of digital technology such as mobile phones for disseminating HPV vaccination reminders in different languages to migrants [56]. In Australia, Denmark, and the USA, electronic health records enabled retrospective cohort studies to investigate HPV vaccination among various migrant populations [68,71,73]. In Denmark, the Danish National Health Register maintains national records of all public healthcare services that utilise government revenue [68]. In Nebraska, USA, electronic medical records exist with refugee health data, captured upon entry and updated post-entry, and this proved useful in examining determinants of HPV vaccination among Burmese refugee girls resettled in Nebraska [71]. In Queensland, Australia, data from an electronic health database was utilised to determine catch-up vaccination processes and determinants of under-immunisation among refugees resettled in East Queensland [73].

## 13. Health System Determinants of HPV Vaccination Uptake

The 5As framework [41] was utilised to explore health system software-related determinants of HPV vaccination uptake (Table 5).

Ease of access enables vaccine uptake, for instance, targeted interventions such as refugee health programmes at point of entry in the USA [69,71] and polyclinics catering for vulnerable persons regardless of legal status in Greece [66], which provided easily accessible and convenient access to health services (including HPV vaccination) for migrant populations. Sometimes, legal status is a barrier to accessing care as observed in Denmark, where asylum seekers whose legal status is yet to be determined are not eligible for preventive care services, including HPV vaccination [68]. A common sentiment is that language barriers may impede access to available information [52,53,67]. Language emerged as a significant barrier to accessing cervical cancer prevention services, including HPV vaccination, among Middle Eastern refugee women in Türkiye [67], while, in the USA and Canada, HPV-related information materials in English deterred access to migrants not proficient in the language [52,53]. When linguistic barriers impeded access to available information, some migrant parents relied on their children’s knowledge and understanding of HPV-related information to guide decision making, for example, USA-based refugees from Cambodia, sub-Saharan Africa, and the Middle East [52,64], and the Netherlands [57]. Among a sample of Syrian refugees residing in Greece, about one third of refugees in one study explained that they would not know where to locate a doctor if they needed healthcare due to unfamiliarity with Greek language [60]. Navigating unfamiliar healthcare systems in host countries, which was compounded by language barriers, also impeded migrants’ access to HPV vaccination as observed in a study of Cambodian refugees resettled in the USA [50].

Affordability is linked with access given that cost may either enable or impede access to and, consequently, uptake of vaccination services. Approximately 92% of a sample of Bhutanese refugees in Nepal indicated willingness for their daughters to receive HPV vaccination if it were free [59], while close to 60% of a sample of unvaccinated refugee and migrant women in Ohio, USA, indicated willingness to get vaccinated if the HPV vaccine was offered free or at a reduced cost [61]. A contrasting view was documented in a Canadian study of newcomer migrants, some of whom were willing to pay for HPV vaccination in instances where they were ineligible for free vaccines [75]. In this regard, willingness to vaccinate was determined by whether the vaccine was available free or at a fee. Willingness to vaccinate may also be considered an indicator of acceptance.

Awareness and acceptance, both closely intertwined, were the most identified determinants of HPV vaccination uptake. On one hand, a study on HPV vaccination among American Cambodian teenagers, of whom 70% were refugees and migrants, showed higher odds of HPV vaccination (OR = 4.08; 95% CI: 1.50–11.05) among daughters of mothers who were knowledgeable about HPV [64]. On the other hand, despite HPV vaccination being freely available in Italy, 44% of migrants and refugees in one study acknowledged not being aware of such services [65]. Similarly, a qualitative study exploring cervical cancer screening and prevention practices of refugee women in San Diego, California, observed that, though HPV vaccines are freely available, 17 of the 18 women interviewed were not aware of the vaccine, and none of their children were vaccinated [52]. Lack of awareness may hinder vaccine acceptance and uptake as observed among a sample of Middle Eastern refugees in Melbourne, Australia [58]. Low risk perception due to limited HPV-related awareness was associated with low HPV vaccination uptake among immigrant and refugee catch-up groups in a Canadian Province [54]. In addition to low awareness, Bhutanese refugee women’s health and wellbeing were shaped by social determinants, linked to prolonged residence in refugee camps, which increased their risk of HPV infection and cervical cancer, including early sexual debut, multiple partners and marriages, alcohol use, and smoking [59].

Culturally appropriate packaging of health information is also a determinant of awareness and acceptance, as observed among a sample of Somali refugees resettled in the Netherlands who preferred oral communication in their home language to printed information in English or Dutch [57]. The use of visual health information messages to bridge language gaps was recommended in a study of USA-based refugees from sub-Saharan Africa and the Middle East [52]. In certain instances, misinformation was circulated via informal sources of information such as interpersonal networks comprising family and friends, and the media (including social media). For instance, media sources yielded amorphous information on HPV vaccination among a sample of USA-based migrants and refugees [74]. Reliance on (mis)information sharing via interpersonal lay networks, among a sample of migrant and refugee women in Australia and Canada, led to the belief that HPV vaccination causes cancer [55], while others in the USA thought that HPV infections occurred due to poor hygiene and consuming spoiled food in refugee camps [64]. Misinformation may hinder acceptance. Culturally appropriate packaging of health information includes consideration of the forums where such information is disseminated, suggesting a shift from health facilities to community-based spaces tailored to linguistic and cultural specifications, as exemplified in a USA-based and a Canadian study [53,64].

The framing of HPV-related messages may also influence acceptance and uptake of HPV vaccination. Among USA-based Cambodian refugee mothers, framing HPV vaccination as protective enabled acceptance because it is consistent with the historical/political narrative of mothers protecting daughters from harm during the Khmer Rouge genocide in Cambodia (1975–1979) and subsequent forced migration to refugee camps in Thailand, before eventually being resettled in the USA [50]. The belief that cervical cancer is a ‘Western’ disease may facilitate HPV vaccine uptake by increasing perceived risk among resettled refugees. This was observed in a study of sub-Saharan African and Middle Eastern refugees resettled in the USA [52]. Likewise, the perception of HPV vaccine as (i) protection from cervical cancer and (ii) similar to other routine vaccinations, promoted acceptance among some Somali refugee women in the Netherlands [57]. Even so, mistrust in government intentions linked to war-related experiences, hindered acceptance as observed among this group of Somali refugee women, some of whom expressed fear and misconceptions of being experimental objects to monitor long-term effects of HPV vaccination [57]. Acceptance among some migrant groups was impeded by concerns about the long-term effects of the vaccine and whether it was effective in curbing HPV infections [51,63].

Links were posited between migrants’ duration of stay in host countries and increased uptake of HPV vaccination. Higher uptake, attributed to acculturation, was observed in a qualitative study of refugees and migrants who had lived in Canada for over five years compared to those who had been in Canada for less than five years [54]. In Denmark, longer duration of stay and higher parental education were linked to higher odds of HPV vaccination among Asian, Bosnian-Herzegovinian, and Stateless Palestinian refugee girls compared to local Danish girls [72]. A contrasting trend was observed in Queensland, Australia, where refugees and asylum seekers who arrived in Australia prior to 2010 were more likely to be under-immunised compared to their recently resettled counterparts [73]. A possible explanation for this variance is the likelihood that uptake may have increased after Queensland Government endorsed the Refugee Health and Wellbeing Policy and Action Plan (2017–2020) [73]. Prevailing socio-cultural and religious attitudes and beliefs hindered awareness and acceptance of HPV vaccination in some migrant communities. These include cultural taboos prohibiting sex-related conversations between parents and daughters, conflation of HPV vaccination with endorsing pre-marital sex in cultures where girls’ virginity is lauded as virtuous, fear of the vaccine causing infertility, and fear of ostracization of errant girls reported among involuntary migrants residing in the Netherlands and Canada [56,57]. Three studies observed that the belief that their daughters were too young to be sexually active and, consequently, low perceived risk of HPV infection impeded migrant parents’ acceptance of the vaccine [53,57,63].

The significant role of recommendations by health professionals in facilitating activation, in the sense of positively influencing caregivers’ intent to vaccinate their children and/or nudging them to vaccinate their children, was a recurring theme. The high likelihood of heeding health professionals’ recommendations was linked to trust in the healthcare system and health professionals involved in service delivery therein. This was reported in studies conducted among involuntary migrants resettled in the USA [50,52,71], Australia [58], and among mixed groups of voluntary and involuntary migrants resettled in Canada [54,56,75]. In a qualitative study of facilitators and barriers to cervical cancer screening and HPV vaccination, 27 out of 31 women ranked doctors’ recommendation as the top activation factor with regard to influencing decisions to vaccinate their children [49]. Where there was a lack of health provider recommendation, this was identified as a barrier to uptake of HPV vaccination as demonstrated among a small sample of Cambodian teenage refugees residing in the USA [64]. Despite the Health Care Consent Act which permits adolescents in Ontario to obtain HPV vaccination without parental consent, health providers were reticent to vaccinate adolescents due to the possibility that parents may not approve of this [56], which may deter willing adolescents from receiving the vaccine. The role of family support, and particularly in patriarchal contexts, featured in two studies, one in which women refugees noted the supportive role of their husbands in HPV vaccination-related decision making for their children [49]. The other study mentioned gender-related diminished agency and freedom, linked to patriarchy, as the context in which refugees and migrants navigate sexual and reproductive health-related issues, including HPV vaccination [55]. In contrast, refugee mothers’ disapproval of HPV vaccination in the Netherlands influenced rejection of the vaccine by their daughters [57], implying a sense of agency among these mothers. The same study mentioned that some refugee women thought HPV vaccination was compulsory in which case compliance may activate vaccination [57].

Activation may also be impeded by under-prioritisation of preventive care. It is worth noting how the lack of preventive care services (e.g., HPV vaccination) in migrants’ home countries could contribute to under-prioritisation of preventive care among migrants themselves post-resettlement, as observed in studies of refugee women resettled in USA [52] and Australia [58] and refugee and migrant women in Canada and Australia [55]. Under-prioritisation was also featured in a study of USA-based Cambodian mothers which alluded to the possibility that survival instinct, associated with forced displacement and resettlement, may lead to under-prioritisation of preventive health services such as HPV vaccination [50]. In this study, under-prioritisation of preventive care was implied in some refugee women’s view that it was unnecessary to fix non-existent problems [50]. The use of incentives in facilitating activation was mentioned in the ‘no jab no pay’ policy implemented in Queensland, Australia, in 2016, through which government incentives including childcare and family tax benefits were offered to parents of fully immunised children as per the NIP schedule or an accepted catch-up schedule [73]. This policy resulted in a marked increase in full immunisation and a decrease in under-immunisation, which may positively impact HPV vaccination particularly in Queensland where it is one of the immunisations with the lowest uptake among refugees and asylum seekers [73].

## 14. Health System Performance Indicators and Reported Considerations for Migrant-Inclusive HPV Vaccination Services

Indicators of robust health systems contained in the Witter et al. Health System Strengthening Framework [44] were mapped alongside practice, provider and patient-level influences of the P3 Model [40] and determinants of HPV vaccination delivery and uptake to illustrate the complex, dynamic, interdependent relationships and consequent health outcomes among the various health system components (Table 6).

Equity was featured in the context of leadership and governance, specifically in policy adaptation and implementation to facilitate inclusion of migrants. At practice level, inclusion of migrants in NIPs in several countries is evidence of progress towards equitable immunisation service delivery. At provider level, variances in policy implementation have resulted in persistent inequity in access to immunisation services among certain sub-populations of migrants such as involuntary migrants whose access to vaccination services may be dependent on one’s legal status in the host country of residence, for instance, in asylum seekers in Denmark [68]. At patient level, equity translates to if and how readily migrant populations can access HPV vaccination and the affordability of these services, which determines uptake. Equity is a central thread interwoven across other performance indicators in the sense that quality, resource mobilisation, high immunisation coverage, social and financial risk protection, and responsiveness are all contingent on equitable service delivery and uptake.

Quality indicators evident in HPV vaccination service delivery at practice and patient levels include updated and synchronised electronic health databases with migrants’ immunisation records, which were not available in many countries despite the absence of records being a commonly shared concern raised by host countries, including Canada [56] and Lebanon [63]. The absence of accurate records may result in under- and/or -over-immunisation, as observed in a Canadian study [56] and, consequently, increased risk of VPDs among the under-immunised and wastage of resources in the case of over-immunisation. At provider level, public–private partnerships between governments and NGOs may boost quality by enhancing service delivery and consolidating resources (Table 6), as demonstrated by the polyclinics model in Greece [66] and the refugee clinic model in Queensland, Australia [73].

Resource mobilisation is exemplified in the various HPV vaccination programmes available and how this pans out at practice, provider, and patient levels. These include school-based programmes, the most utilised avenue for HPV vaccination and supplementary catch-up vaccination. Facility-based programmes are relatively widely available in most host countries including the USA [50,71], Denmark [68,72], Greece [66], and Australia [73], while Canada offers both school-based and facility-based HPV vaccination programmes. In contrast, catch-up vaccination is available in fewer countries such as Australia [58,73], Canada [53,54], and Denmark [68,72]. Sometimes certain sub-populations of migrants are ineligible for free catch-up vaccination, for instance, refugees and migrants aged 16–27 years old in Ontario, Canada [75].

High immunisation coverage is contingent on achieving equity, quality, resource mobilisation, social/financial risk protection, and responsiveness goals at practice, provider, and patient levels. Likewise, social and financial risk protection, which is linked with health financing, is contingent on achieving equity and responsiveness goals at practice, provider, and patient levels.

Responsiveness occurs in the context of interactions among health system actors including health providers, policy makers, individual patients, families, and communities. During these interactions, needs and expectations are indicators of responsiveness in as far as whether the health system meets the expectations of migrant populations. At practice and patient levels, health information materials produced in languages that are unfamiliar to migrants impede HPV vaccination service delivery and uptake as addressed previously (Table 5). At patient level, this is exacerbated by misinformation circulated via interpersonal networks, with a negative ripple effect on access, awareness, acceptance, and activation as observed among migrant and refugee women in Australia, Canada [55], and the USA [64]. At provider and practice levels, health provider recommendations are an exemplar of responsiveness to migrants’ need for HPV vaccine endorsement from trusted sources, which is likely to increase uptake and, conversely, decrease uptake if health providers fail to recommend the vaccine to migrants (Table 5). At patient level, two additional responsiveness-related impediments are mistrust of host country governments, for example, among Somali refugee women in the Netherlands [57], and under-prioritisation of preventive care which may be partially attributed to experiences in home countries and/or migration experiences, as exemplified among a group of USA-based refugee women [52] and a group of refugee and migrant women residing in Canada and Australia [55].

## 15. Discussion

This review demonstrates efforts to include involuntary migrants in NIPs through policy adaptation at regional and national levels and by working to ensure access to immunisation for all, including involuntary migrant populations who are deemed vulnerable [33]. To the best of our knowledge, this is one of the first comprehensive systematic reviews that has focused on involuntary migrants and their experiences with HPV vaccination. This adds to the strong body of evidence on the importance of HPV vaccination for all migrant populations regardless of who they are, where they reside, or their legal status. Two previous reviews studied determinants of HPV vaccination among migrants as a homogenous group [76,77], while a third review examined health system determinants of HPV and MMR vaccination among disadvantaged, minority, or underserved populations [78]. One of these three reviews took note of the varied terms used to describe migrants and acknowledged that, in not focusing on diverse sub-populations of migrants, one could miss out on nuances akin to specific sub-populations [70].

There were significantly fewer studies from LMICs compared to HICs. This reiterates the findings of recent reviews addressing HPV vaccination among migrant populations in which the evidence was largely from HICs but based on migrant populations from LMICs [76,77,78]. A plausible reason for this disparity is that there has been slower adoption of HPV vaccine in routine programmes in LMICs compared to HICs for various reasons, including financial constraints, health system barriers, technical limitations, and performance gaps between first and second dose completion, with high dropout rates in the latter [2,9,13]. This comparatively slower adoption may partially account for the dearth of publications on HPV vaccination among migrant populations in LMICs. Notwithstanding, our findings suggest that there is still an argument to be made for understanding HPV vaccination-related needs of involuntary migrant populations living in LMICs. For instance, Nepal-based Bhutanese refugee women’s knowledge of HPV as a cause of cervical cancer and awareness of HPV vaccination was much lower than that of local Nepalese women residing in the same district [59]. The situation was further compounded by other social determinants such as early marriage and sexual debut, multiple partners and marriages, which placed them at higher risk of HPV infection and cervical cancer compared to their Nepalese counterparts [59]. In Lebanon, low uptake (1.5%) of HPV vaccination among Syrian refugee girls was associated with low HPV vaccine-related knowledge among mothers [63]. We propose that more studies be conducted among involuntary migrant populations living in LMIC contexts given that this is where most are hosted.

Key findings from this review, which seem apparent in different kinds of health system settings, include common access-related barriers related legal status and lack of clear policies, awareness-related barriers linked to communication challenges akin to language, literacy and culturally appropriate health messaging, and acceptance-related barriers associated with sociocultural attitudes, beliefs and practices coupled with misinformation and distrust of host country governments, health systems and health providers. These findings are not limited to barriers to HPV vaccination among involuntary migrants but cut across a broad range of routine vaccines including measles–mumps–rubella, diphtheria–pertussis–tetanus, poliomyelitis, pneumococcal conjugate, Haemophilus influenzae type b, hepatitis B, and COVID-19 vaccines [18,20,21,79,80,81,82], and across lifespan, including childhood and adolescence [18,79,80,81,82], as well as adulthood [20,21]. Further, the mentioned barriers to vaccination were reported among mixed sub-populations of migrants [20,21,81,82] and also specifically among involuntary migrants [18,79,80], which renders HPV a relevant proxy for examining the determinants of vaccination delivery and uptake among migrant populations and their sub-populations.

Among involuntary migrant populations, the role of sociocultural norms, beliefs, and practices as protective factors emerged as key influencers of HPV vaccination uptake. On one hand, framing HPV vaccine as protective encouraged refugee mothers to vaccinate their daughters, as demonstrated in the case of Cambodian refugees resettled in the USA, because this narrative is consistent with the socio-political and historical context of protecting their children during war [50]. On the other hand, sociocultural norms deemed protective, for instance, abstinence from pre-marital sex, were associated with reticence to accept HPV vaccination among involuntary migrant parents, caregivers, and guardians of adolescent girls [51,57,62]. A review by Tankwanchi et al. on vaccine hesitancy among migrant communities [27] elucidates the link between sociocultural factors and HPV vaccine uptake among migrants. Based on the 3Cs of vaccine hesitancy (complacency, confidence, and convenience) [83]. Tankwanchi et al. explain complacency in terms of low perceived risk of disease, attributed to the protective role of migrants’ sociocultural attitudes and beliefs, culminating in refusal or delays in accepting and receiving the vaccine [27].

Our specific focus was on involuntary migrants. Despite some similarities between the experiences of migrant populations as a collective and those of involuntary migrants as a sub-population, factors related to the disruptive and traumatic context of forced migration present some unique challenges to HPV vaccination delivery and uptake among involuntary migrants. These include sudden forced displacement, damage to healthcare infrastructure, and unavailability of health professionals during conflict and other humanitarian emergencies resulting in missed vaccination opportunities, loss and/or damage to vaccination records, and increased risk of VPD outbreaks in holding camps [18,79,84,85]. Against this background, three key determinants of vaccination among involuntary migrants relating to equity, resource mobilisation, and responsiveness emerged from our study and are reiterated in the extant literature.

(In)equity in access to HPV vaccination featured among involuntary migrants. For instance, in Lebanon where the HPV vaccine is not available free via routine NIPs in the public sector, the out-of-pocket cost of obtaining the vaccine from the private health sector deterred Syrian refugees from accessing HPV vaccination [63]. In this regard, routinisation of HPV vaccines may enhance access by making these vaccines available at no or low cost to this population. Despite free HPV vaccine availability, lower HPV vaccination rates among involuntary migrants from LMICs settled in the USA relative to the local population in host countries [52,64]—attributed to limited/lack of knowledge—point to structural inequity. Structural inequity faced by involuntary migrants resurfaced during COVID-19 in the context of human rights-related access barriers, including failure to prioritise vaccination of involuntary migrants, imposition of legal barriers, and fear of being caught, leading to avoidance of healthcare facilities and anti-migrant discrimination [26,86]. In two instances in the USA, where specific policies and strategies facilitated equitable access to health services by involuntary migrants, higher uptake of the first dose of HPV vaccine was reported among involuntary migrants compared to the general population in Nebraska and Massachusetts [69,71], underscoring the significance of equity in HPV vaccination access.

Resource mobilisation has been successfully applied in integration of vaccination services with other services, including integrating childhood immunisation and nutritional services [87], child immunisation with maternal preventive health services, and HPV vaccination with sexual and reproductive health services [88], during humanitarian emergencies. Integrated interventions may also contribute to improving the quality of services. Resource mobilisation also includes catering for missed vaccination opportunities by offering supplementary catch-up vaccination, which has featured more in routine childhood vaccination among involuntary migrants [18] than in HPV vaccination, where catch-up services are only available at no cost in some HICs such as Australia [58,73].

Applying a health systems lens allowed for exploration of health system responsiveness [89], by showcasing how NIPs have adapted their vaccination policies to include migrants, highlighting the needs and expectations of migrant populations, and, further, how health systems have responded to migrants’ needs and how this has shaped vaccine uptake. In this regard, a health systems’ perspective was beneficial in demonstrating the role of various health system components in facilitating and boosting vaccine delivery and uptake in this population [90,91]. Vaccination also emerged as a potential avenue for health systems strengthening in the context of vaccination-related policies and interventions that cut across building blocks, which mutually influence each other over time, as indicators of strong health systems [44,92]. Responsiveness may be a challenge at provider level in situations where health infrastructure is damaged and health professionals may be unavailable to deal with preventive services, which may impede sharing of health-related messages.

## 16. Recommendations

**Figure 7:**
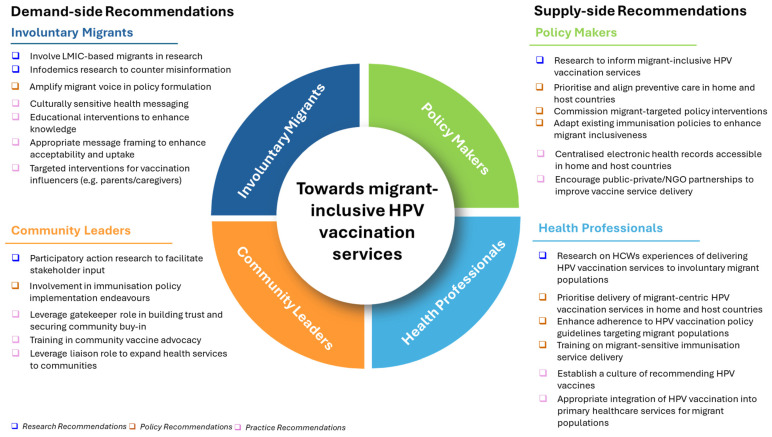
Recommendations for key health system actors.

Our findings have important implications for key actors working in the immunisation, health systems, and migrant health space. These may be considered across demand and supply sides. Several actors need to be held accountable in both demand and supply spaces. We highlight recommendations for research, policy, and practice for four key health system actors: involuntary migrants and community leaders, on the demand side, and policy makers and health professionals, on the supply side (Figure 7).

On the demand side, we recommend involvement of involuntary migrants and community leaders, including those in LMICs, in research to better understand their needs and existing gaps, in addition to research targeting infodemics to counter HPV vaccine-related misinformation. At policy level, inclusion of involuntary migrants in policy formulation and involving community leaders in HPV vaccination-related policy implementation will aid in amplifying their voices in vaccination-related agenda setting and execution. At practice level, specific attention should be directed toward alleviating communication barriers among involuntary migrants, coupled with targeted interventions to enhance knowledge and awareness and ultimately, to secure buy-in of HPV vaccination influencers including parents and guardians. The gatekeeper and liaison roles of community leaders should be leveraged for building trust and facilitation of training in community vaccine advocacy.

On the supply side, policy makers should conduct more policy-relevant research at macro-, meso-, and micro-levels to inform involuntary migrant-inclusive HPV vaccination services. Further, there is need for more research exploring health professionals’ experiences of delivering HPV vaccination services to involuntary migrant populations. At policy level, we recommend that policy makers prioritise preventive care and involuntary migrant-targeted interventions such as catch-up HPV vaccination. Policy makers have a critical role to play in adapting existing immunisation policies, to ensure that none are inadvertently excluded from accessing HPV vaccination services either on legal or financial grounds. At policy level, health professionals should prioritise delivery of migrant-centric HPV vaccination services, which necessitates training in migrant-sensitive immunisation service delivery. At practice level, there is need for concerted effort among policy makers and health professionals to bridge HPV vaccination service delivery gaps through strategies such as encouraging public–private partnerships by policy makers and appropriate integration of HPV vaccination with primary healthcare services for migrant populations by health professionals.

## 17. Limitations

Majority of the studies sampled (24/27) were from the experiences of migrants resettled in HICs where most HPV vaccination programmes are concentrated, as noted in several studies [9,13,76,93], thus may not be fully representative of LMIC contexts. Even so, evidence from HICs may be useful in the following: (i) understanding immunisation contexts in LMICs, which are the home countries to a large population of migrants resettled in HICs, and (ii) informing immunisation policies for migrant populations in high-income and other contexts, particularly LMICs where close to 76% of forcibly displaced migrants reside [29]. Further, focusing solely on HPV vaccination was topically limiting. However, in the context of qualitative evidence synthesis coupled with an analytical model comprising four frameworks, the detailed narrative data was sufficient for rich and in-depth analysis, leading to the results presented.

## 18. Conclusions

Prioritising vaccination programmes linked with non-outbreak-related childhood and adolescent diseases is challenging in the context of humanitarian crises given fragile health systems, limited resources, loss of health infrastructure, and deployment of health staff to respond to emergency health issues, all of which sideline preventative services like HPV vaccination programmes. The implication is that relevant actors at all levels of the health system will need to carefully consider trade-offs while ensuring that involuntary migrant adolescent girls living in these contexts or resettled in host countries are not left at risk of developing cervical cancer.

## Figures and Tables

**Table 1:** Summary of inclusion and exclusion criteria.

Selection Criteria	Inclusion Criteria	Exclusion Criteria
**Population**	Involuntary migrants/forcibly displaced	Studies that do not include involuntary migrants either as the main population or as a sub-population of migrants
**Intervention**	HPV vaccination	Other vaccines recommended for adolescents including Tetanus, Diphtheria, and Pertussis (Tdap) booster, Hepatitis B, and Rubella, unless HPV vaccination was included in the regimen of vaccines reported in a study
**Comparison**	N/A	N/A
**Outcomes**	Health system determinants of delivery and uptake of HPV vaccination services among involuntary migrant populations	Studies that do not include evidence on HPV vaccination service provision-related supply- and demand-side factors
**Study design**	Peer-reviewed qualitative, quantitative, and mixed-methods empirical studies written in English language	Secondary studies (including reviews), opinions, perspectives, and commentaries

**Table 2:** Analytical framework—health systems determinants of vaccination delivery and uptake among migrant populations.

**Context**			
▪ Forced displacement/involuntary migration, e.g., due to war, persecution, humanitarian crises. ▪ Global goals/policies: Immunisation Agenda 2030; WHO 2022 Global Evidence Review on Health and Migration; Universal Health Coverage.			
**5As framework domains**	**WHO health system building blocks**	**Indicators of robust health systems**	**P3 model—health system influences**
▪ access	▪ service delivery	▪ equity	▪ provider level
▪ awareness	▪ medicines/vaccines	▪ quality	▪ patient level
▪ acceptability	▪ information systems	▪ resource mobilisation	▪ practice level
▪ acceptance	▪ finance	▪ high immunisation coverage	
▪ activation	▪ health workforce	▪ social/financial risk protection	
	▪ leadership/governance	▪ responsiveness	

**Table 3:** Descriptive characteristics of the 27 included studies.

Author/Year	Design	Classification of Migrants	Home Countries/Region(s)	Host Countries	Actors, e.g., Providers, Patients, Caregivers
Allen et al., 2019 [49]	Qualitative	Refugees	Somalia	USA	mothers
Badre-Esfahani et al., 2020 [68]	Cohort	Involuntary vs. voluntary migrants	Central Asia, SE Asia, SSA, Latin America, Western countries; Turkey, Iraq, Lebanon, Pakistan, Afghanistan, Somalia, Iran, Morocco	Denmark	women
Berman et al., 2017 [69]	Cohort	Refugees	Predominantly Iraq, Bhutan, Somalia, and other SSA countries	USA	adolescent males and females (9–26 years)
Bhatta et al., 2020 [59]	Cross-sectional	Refugees	Bhutan	Nepal	women
Burke et al., 2015 [50]	Qualitative	Refugees	Cambodia	USA	mothers
Dalla et al., 2022 [60]	Cross-sectional	Refugees	Syria	Greece	women
Davidson and Fisher, 2025 [58]	Qualitative	Refugees and asylum seekers	Myanmar, Iraq, Syria, Iran, Sri Lanka, Colombia, Indonesia, Lebanon, Malaysia, Togo, Pakistan	Australia	women
Do et al., 2009 [51]	Qualitative	Refugees/migrants	Cambodia	USA	parents and community leaders
Elmore et al., 2021 [70]	Cohort	Refugees	Afghanistan, Bhutan, Burma, ColombiaDR Congo, El Salvador, Eritrea, Iran, Iraq, Moldova, Nepal, Syria, Russia, Sudan, Syria, Ukraine	USA	women
Gebre et al., 2021 [61]	Cross-sectional	Refugees/migrants	Somalia and Mexico	USA	women
Ghebrendrias et al., 2021 [52]	Qualitative	Refugees	Sudan, Somalia, Kenya, Ethiopia, Eritrea, Congo, Uganda, Syria, Iraq, Egypt, and Morocco	USA	women
Kenny et al., 2021 [71]	Cohort	Refugees	Burma	USA	adolescent females (11–26 years)
Kepka et al., 2018 [62] (see Lai sequel study)	Mixed Methods	Refugees	Burundi, Congo, Rwanda, Liberia, Tanzania	USA	parents, legal guardians, caregivers
Khan et al., 2023 [53]	Qualitative	Migrants including refugees	Refugees from West Asia; migrants from South and Southeast Asia	Canada	parents
Kmeid et al., 2019 [63]	Cross-sectional	Refugees	Syria	Lebanon	parents and legal guardians
Lai et al., 2017 [74] (see Kepka sequel study)	Mixed Methods	Refugees	Burundi, Congo, Rwanda, Liberia, Tanzania	USA	parents, legal guardians, caregivers
Lee et al., 2016 [64]	Cross-sectional	Refugees	Cambodia	USA	mothers
McComb et al., 2018 [54]	Qualitative	Immigrants including refugees	Africa, Asia, South America	Canada	women (16–26 years old)
Metusela et al., 2017 [55]	Qualitative	Refugees/migrants	Afghanistan, Iraq, Somalia, South Sudan, Sudan, Sri-Lanka (Tamil), India (Punjabi), South America (Latina), Sudan	Canada and Australia	women
Moller et al., 2018 [72]	Cohort	Refugees	55	Denmark	adolescent females
Napolitano et al., 2018 [65]	Cross-sectional	Refugees/immigrants	mainly SSA (64.5%), Eastern Europe, South Asia, North Africa, South America, Central Asia	Italy	adolescent females (12–26 years) and parents
Nyanchoga et al., 2021 [73]	Cohort	Refugees and asylum seekers	42 countries—listed ones: Middle East (Afghanistan, Iran, Iraq); Asia (Myanmar, India, Pakistan, Sri Lanka); SSA (DRC, Eritrea, Ethiopia, Kenya, Somalia, Sudan); Papua New Guinea, Solomon Islands	Australia	children, adolescents, and adults
Riza et al., 2020 [66]	Cross-sectional	Involuntary vs. voluntary migrants	Middle East incl. Syria, Afghanistan, and Iran; SSA incl. Nigeria, Ethiopia, Cameroon, and Kenya; Eastern European countries incl. Albania, Bulgaria, and Georgia	Greece	women
Rubens-Augustson et al., 2019 [56]	Qualitative	Immigrants including refugees	Not given	Canada	health providers
Salad et al., 2015 [57]	Qualitative	Refugees	Somalia	Netherlands	women
Snoubar et al., 2025 [67]	Cross-sectional	Refugees	Iraq, Palestine, Syria, Yemen	Türkiye	women
Wilson et al., 2021 [75]	Mixed Methods	Immigrants including refugees	SSA (36%); MENA (58%); Other (6%)	Canada	adolescents (16–27 years) and caregivers

SSA—Sub-Saharan Africa; MENA—Middle East and North Africa; Migrants and immigrants used interchangeably.

**Table 4:** Health system determinants of HPV vaccination service delivery based on WHO building blocks.

Building Blocks	Enablers (+)	Impediments (−)
1. Leadership/governance	1.1 policies prioritising migrants’ health needs	1.1(a) HPV vaccination policies not implemented in some countries
	1.2 decentralised governance and variations in immunisation policy implementation	1.2(a) decentralised governance and variations in immunisation policy implementation
	1.3 governments as gatekeepers in migrant-inclusive immunisation policy implementation	
2. Service delivery AND medicines/vaccines	2.1 school-based HPV vaccination programmes	2.1(a) no HPV vaccination programme available
	2.2 supplementary catch-up vaccination	2.2(a) HPV vaccination available in NIP but as voluntary routine not mandatory routine vaccination
	2.3 migrant-targeted interventions	2.3(a) health messaging targets limited audience
	2.4 integrated services	2.4(a) limited access, e.g., schools, holding camps, eligibility based on legal status
	2.5 public–private partnerships	
3. Health workforce	3.1 health provider recommendation	3.1(a) no health provider recommendation
	3.2 health provider main source of HPV vaccination-related information	3.2(a) health provider time constraints—limited time to discuss HPV vaccination
	3.3 vaccine administration (including consent)	3.3(a) health provider reticence to recommend vaccination
		3.4(a) health provider inadequately trained to serve migrant populations
4. Health information systems	4.1 electronic health databases with migrants’ records (including immunisation data)	4.1(a) no vaccination records available for migrant populations
		4.2(a) no centralised or synchronised electronic databases with migrants’ immunisation data
5. Financing	5.1 HPV vaccination free for all (including migrants) via NIPs and other support programmes	5.1(a) cost for ineligible, partially covered and uninsured migrants

**Table 5:** Summary of health system determinants of HPV vaccination uptake featured, based on 5As Framework.

Determinants of Uptake (5As)	Enablers (+)	Impediments (−)
1. Access	1.1 easy access/convenience	1.1(a) legal status
	1.2 navigating language barriers	1.2(a) unfamiliarity with host country’s healthcare system
		1.3(a) language barriers
2. Affordability	2.1 free vaccination	2.1(a) cost-prohibitive
	2.2 willingness to vaccinate	
3. Awareness and Acceptance	3.1 adequate knowledge about HPV vaccination: ▪ culturally appropriate health promotion materials and forums ▪ information sources	3.1(a) low/lack of knowledge about HPV vaccination: ▪ language barriers ▪ misinformation ▪ mistrust of governments’ intentions ▪ living conditions
	3.2 framing/perception of HPV vaccination: ▪ protective and/or preventive ▪ a western disease	3.2(a) concerns about long-term effects and effectiveness of vaccine
	3.3 length of stay in host country	3.3(a) length of stay in host country
		3.4(a) sociocultural and religious attitudes, beliefs, and practices ▪ sex deemed a taboo topic ▪ allowing HPV vaccination is endorsing pre-marital sex and promiscuity ▪ young girls are not sexually active ▪ preference for traditional medicine
4. Activation	4.1 health provider recommendation	4.1(a) health provider reticence to recommend HPV vaccination
	4.2 women’s agency and family support	4.2(a) mothers’ disapproval
	4.4 assumption that HPV vaccination is compulsory	4.4(a) preventive care not prioritised
	4.5 incentives	

**Table 6:** Health system performance indicators, influences, and determinants of HPV vaccination.

Health System Performance Indicators	Practice-Level Influences	Provider-Level Influences	Patient-Level Influences	WHO Building Blocks/Delivery Determinants	5As of Uptake
**Equity**	Delivery: *enabler*—policy adaptation and implementation to include migrants	Delivery: *enabler*—policy implementation to include migrants *impediment*—differential implementation (exclusion of certain migrant sub-populations)	Uptake: *enabler*—easy, convenient, free access *impediments*—access contingent on legal status, language, knowledge/awareness-related barriers	Leadership/governance/ policy	Access Affordability
**Quality**	Delivery: *enabler*—updated, synchronised electronic health databases with migrants’ immunisation records *impediment*—no records of migrant immunisation data	Delivery: *enablers*—public–private partnerships *impediments*—health provider time constraints, limited/lack of training, reticence to recommend HPV vaccine	Delivery: *impediments*—no records of migrant immunisation data (could result in under- and/or over-immunisation)	Health information systems Service delivery Medicines/vaccines	Access Awareness
**Resource mobilisation**	Delivery: *enablers*—school-based programmes, supplementary catch-up, migrant-specific interventions, integrated services	Delivery: *enablers*—school-based programmes, supplementary catch-up, migrant-specific interventions, integrated services	Delivery: *enablers*—school-based programmes, supplementary catch-up, migrant-specific interventions, integrated services	Service delivery Medicines/vaccines	Access
**High immunisation coverage**	Delivery: *enablers*—public–private partnerships Uptake: *enablers*—culturally appropriate health messaging	Delivery: *enablers*—public–private partnerships, health provider recommendations; *impediments*—no health provider recommendation	Uptake: *enablers*—easy, convenient, free access, health provider recommendation, incentives *impediments*—difficult to access, socio-cultural beliefs	Service delivery Medicines/vaccines Health workforce	Access Awareness Acceptance Activation
**Social/financial risk protection**	Delivery and uptake: *enabler*—free HPV vaccine regardless of legal status *impediment*—HPV vaccine cost partially covered or at own cost	Delivery and uptake: *enabler*—free HPV vaccine regardless of legal status *impediment*—HPV vaccine cost partially covered or at own cost	Delivery and uptake: *enabler*—free HPV vaccine regardless of legal status *impediment*—HPV vaccine cost partially covered or at own cost	Health financing	Affordability Access Awareness
**Responsiveness**	Delivery: *impediment*—health promotion materials in English are not understood	Uptake: *enablers*—health provider recommendations, framing HPV vaccination as protective *impediments*—no health provider recommendation, limited/lack of training, reticence to recommend HPV vaccine	Uptake: *enablers*—health provider recommendations, framing HPV vaccination as protective Uptake: *impediments*—language barriers, mistrust of host country governments, misinformation, no health provider recommendation, under-prioritisation of preventive care	Service delivery	Access Awareness Acceptance Activation

## Data Availability

All data generated or analysed during this review are included in this published article and its supplementary information files.

## Supplementary Materials

The following supporting information can be downloaded at the following: https://www.mdpi.com/article/10.3390/vaccines13101064/s1, Data S1: PRISMA Checklist; Data S2: Search Strings.

## Abbreviations

The following abbreviations are used in this manuscript:

HPV	Human Papillomavirus
LMICs	Low-and-Middle-Income Countries
HICs	High-Income Countries
WHO	World Health Organization
NIPs	National Immunisation Programmes
USA	United States of America
PRISMA	Preferred Reporting Items for Systematic Reviews and Meta-Analyses
JBI	Joanna Briggs Institute
RHAP	Refugee Health Assessment Program
