# Supplementary material for: Health System Determinants of Delivery and Uptake of HPV Vaccination Services Among Involuntary Migrant Populations: A Qualitative Systematic Review
Source: Vaccines (Basel). 2025 Oct 18;13(10):1064. doi: 10.3390/vaccines13101064 (PMC12567867; doi:10.3390/vaccines13101064)
Supplement: Supplementary file 1 [file vaccines-13-01064-s001.zip › Data S2_Search Strings_Oct 2025.pdf]

Data S2: Search Strings

PubMed

Search	Query	Items found
#1	(“Transients and Migrants/classification"[Mesh] OR "Transients and Migrants/legislation and jurisprudence"[Mesh])	439
#2	Migrants OR "asylum seekers" OR displaced OR refugee OR transients OR undocumented OR "forcibly displaced" OR resettled OR "persons-of-interest" OR "internally displaced persons" OR irregular OR illegal OR "illegal immigrant" OR aliens OR "foreign-born" OR "internationally displaced person" OR "unaccompanied child*" OR "unaccompanied minor" OR "separated child*" OR "involuntary migrant" OR "stateless person"	734,222
#3	(("Transients and Migrants/classification"[Mesh] OR "Transients and Migrants/legislation and jurisprudence"[Mesh])) OR (Migrants OR "asylum seekers" OR displaced OR refugee OR transients OR undocumented OR "forcibly displaced" OR resettled OR "persons-of-interest" OR "internally displaced persons" OR irregular OR illegal OR "illegal immigrant" OR aliens OR "foreign-born" OR "internationally displaced person" OR "unaccompanied child*" OR "unaccompanied minor" OR "separated child*" OR "involuntary migrant" OR "stateless person")	734,222
#4	("Papillomavirus Vaccines/administration and dosage"[Mesh] OR "Papillomavirus Vaccines/supply and distribution"[Mesh])	3,529
#5	"Human papillomavirus vaccin*" OR "HPV vaccin*" OR "human papillomavirus immuni*"	12,062
#6	(("Papillomavirus Vaccines/administration and dosage"[Mesh] OR "Papillomavirus Vaccines/supply and distribution"[Mesh])) OR ("Human papillomavirus vaccin*" OR "HPV vaccin*" OR "human papillomavirus immuni*")	12,694
#7	"health care provider" OR "health provider" OR "health policy" OR Coverage OR uptake OR quality OR availability OR acceptability OR awareness OR access OR "service provision" OR "service delivery" OR affordability OR "healthcare cost" OR "cost effectiveness" OR "healthcare provision" OR efficiency OR safety OR "financial risk protection" OR equity OR responsiveness OR "health outcomes" OR "health system" OR efficacy	10,028,264
#8	(((("Transients and Migrants/classification"[Mesh] OR "Transients and Migrants/legislation and jurisprudence"[Mesh])) OR (Migrants OR "asylum seekers" OR displaced OR refugee OR transients OR undocumented OR "forcibly displaced" OR resettled OR "persons-of-interest" OR "internally displaced persons" OR irregular OR illegal OR "illegal immigrant" OR aliens OR "foreign-born" OR "internationally displaced person" OR "unaccompanied child*" OR "unaccompanied minor" OR "separated child*" OR "involuntary migrant" OR "stateless person")) AND ((("Papillomavirus Vaccines/administration and dosage"[Mesh] OR "Papillomavirus Vaccines/supply and distribution"[Mesh])) OR ("Human papillomavirus vaccin*" OR "HPV vaccin*" OR "human papillomavirus immuni*"))) AND ("health care provider" OR "health provider" OR "health policy" OR Coverage OR uptake OR quality OR availability OR acceptability OR awareness OR access OR "service provision" OR "service delivery" OR affordability OR "healthcare cost" OR "cost effectiveness" OR "healthcare provision" OR efficiency OR safety OR "financial risk protection" OR equity OR responsiveness OR "health outcomes" OR "health system" OR efficacy)	188
	Exported all 188 references to Endnote	

EBSCOHost

Search	Query	Items found
#1	Migrants OR "asylum seekers" OR displaced OR refugee OR transients OR undocumented OR "forcibly displaced" OR resettled OR "persons-of-interest" OR "internally displaced persons" OR irregular OR illegal OR "illegal immigrant" OR aliens OR "foreign-born" OR "internationally displaced person" OR "unaccompanied child*" OR "unaccompanied minor" OR "separated child*" OR "involuntary migrant" OR "stateless person"	861,744
#2	"Human papillomavirus vaccin*" OR "HPV vaccin*" OR "human papillomavirus immuni*"	20,705
#3	"health care provider" OR "health provider" OR "health policy" OR Coverage OR uptake OR quality OR availability OR acceptability OR awareness OR access OR "service provision" OR "service delivery" OR affordability OR "healthcare cost" OR "cost effectiveness" OR "healthcare provision" OR efficiency OR safety OR "financial risk protection" OR equity OR responsiveness OR "health outcomes" OR "health system" OR efficacy	9,506,200
#4	#1 AND #2 AND #3	180
	80 duplicates were automatically removed during export to EndNote, so 100 articles were exported to EndNote	
	Searched in: Academic Search Premier, Africa-Wide Information, CINAHL, Health Source - Consumer Edition, Health Source: Nursing/Academic Edition, APA PsycArticles, APA PsycInfo	

Web of Science

Search	Query	Items found
#1	Migrants OR "asylum seekers" OR displaced OR refugee OR transients OR undocumented OR "forcibly displaced" OR resettled OR "persons-of-interest" OR "internally displaced persons" OR irregular OR illegal OR "illegal immigrant" OR aliens OR "foreign-born" OR "internationally displaced person" OR "unaccompanied child*" OR "unaccompanied minor" OR "separated child*" OR "involuntary migrant" OR "stateless person"	939,933
#2	"Human papillomavirus vaccin*" OR "HPV vaccin*" OR "human papillomavirus immuni*"	13,474
#3	"health care provider" OR "health provider" OR "health policy" OR Coverage OR uptake OR quality OR availability OR acceptability OR awareness OR access OR "service provision" OR "service delivery" OR affordability OR "healthcare cost" OR "cost effectiveness" OR "healthcare provision" OR efficiency OR safety OR "financial risk protection" OR equity OR responsiveness OR "health outcomes" OR "health system" OR efficacy	10,069,547

#4	#1 AND #2 AND #3	122
	Exported all 122 references to EndNote	

Scopus

Search	Query	Items found
#1	(TITLE-ABS-KEY (migrant* OR "asylum seeker*" OR "displaced person*" OR refugee* OR "transient person*" OR "undocumented person*" OR "forcibly displaced person*" OR "resettled person*" OR "persons-of-interest" OR "internally displaced person*" OR "irregular person*" OR "illegal person*" OR "illegal immigrant*" OR "foreign-born person*" OR "internationally displaced person*" OR "unaccompanied child*" OR "unaccompanied minor*" OR "separated child*" OR "involuntary migrant*" OR "stateless person*"))	170,209
#2	(TITLE-ABS-KEY ("Human papillomavirus vaccin*" OR "HPV vaccin*" OR "human papillomavirus immuni*"))	13,604
#3	(TITLE-ABS-KEY ("health care provider*" OR "health provider*" OR "health polic*" OR "health Coverage" OR "vaccin* uptake" OR "vaccin* quality" OR "vaccin* availability" OR "vaccin* acceptability" OR "vaccin* awareness" OR "vaccin* access" OR "vaccin* service provision" OR "vaccin* service delivery" OR "vaccin* affordability" OR "vaccine healthcare cost" OR "vaccin* cost effectiveness" OR "vaccin* healthcare provision" OR "vaccin*efficiency" OR "vaccin* safety" OR "vaccin* financial risk protection" OR "vaccine* equity" OR "vaccin* responsiveness" OR "vaccin* health outcome*" OR "vaccin* health system*" OR "vaccin* efficacy"))	213,162
#4	#1 AND #2 AND #3	44
	Exported all 44 references to EndNote	
